# Validation of DIGIROP models and decision support tool for prediction of treatment for retinopathy of prematurity on a contemporary Swedish cohort

**DOI:** 10.1136/bjophthalmol-2021-320738

**Published:** 2022-03-11

**Authors:** Aldina Pivodic, Lois E.H. Smith, Anna-Lena Hård, Chatarina Löfqvist, Ana Catarina Almeida, Abbas Al-Hawasi, Eva Larsson, Pia Lundgren, Birgitta Sunnqvist, Kristina Tornqvist, Agneta Wallin, Gerd Holmstrom, Lotta Gränse

**Affiliations:** 1 Department of Clinical Neuroscience, Institute of Neuroscience and Physiology, Sahlgrenska Academy, University of Gothenburg, Gothenburg, Sweden; 2 Department of Ophthalmology, Boston Children's Hospital, Harvard Medical School, Boston, Massachusetts, USA; 3 Institute of Health Care Science, Sahlgrenska Academy, University of Gothenburg, Gothenburg, Sweden; 4 Department of Ophthalmology, Hospital Beatriz Angelo, Loures, Portugal; 5 Neonatal Intensive Care Unit, Hospital São Francisco Xavier—Centro Hospitalar de Lisboa Ocidental, Lisbon, Portugal; 6 CEDOC, Chronic Diseases Research Center, NOVA Medical School – Universidade Nova de Lisboa, Lisbon, Portugal; 7 Comprehensive Health Research Centre (CHRC), NOVA Medical School, Universidade Nova de Lisboa, Lisbon, Portugal; 8 Department of Ophthalmology, Luz Saúde, Hospital da Luz, Lisbon, Portugal; 9 Department of Clinical and Experimental Medicine, Linköping University, Linköping, Sweden; 10 Department of Surgical Sciences/Ophthalmology, Uppsala University, Uppsala, Sweden; 11 Länssjukhuset Ryhov, Jönköping, Sweden; 12 Department of Clinical Sciences, Ophthalmology, Skane University Hospital, Lund University, Lund, Sweden; 13 St. Erik Eye Hospital, Stockholm, Sweden

**Keywords:** diagnostic tests/investigation, retina

## Abstract

**Background/Aims:**

Retinopathy of prematurity (ROP) is currently diagnosed through repeated eye examinations to find the low percentage of infants that fulfil treatment criteria to reduce vision loss. A prediction model for severe ROP requiring treatment that might sensitively and specifically identify infants that develop severe ROP, DIGIROP-Birth, was developed using birth characteristics. DIGIROP-Screen additionally incorporates first signs of ROP in different models over time. The aim was to validate DIGIROP-Birth, DIGIROP-Screen and their decision support tool on a contemporary Swedish cohort.

**Methods:**

Data were retrieved from the Swedish national registry for ROP (2018–2019) and two Swedish regions (2020), including 1082 infants born at gestational age (GA) 24 to <31 weeks. The predictors were GA at birth, sex, standardised birth weight and age at the first sign of ROP. The outcome was ROP treatment. Sensitivity, specificity and area under the receiver operating characteristic curve (AUC) with 95% CI were described.

**Results:**

For DIGIROP-Birth, the AUC was 0.93 (95% CI 0.90 to 0.95); for DIGIROP-Screen, it ranged between 0.93 and 0.97. The specificity was 49.9% (95% CI 46.7 to 53.0) and the sensitivity was 96.5% (95% CI 87.9 to 99.6) for the tool applied at birth. For DIGIROP-Screen, the cumulative specificity ranged between 50.0% and 78.7%. One infant with Beckwith-Wiedemann syndrome who fulfilled criteria for ROP treatment and had no missed/incomplete examinations was incorrectly flagged as not needing screening.

**Conclusions:**

DIGIROP-Birth and DIGIROP-Screen showed high predictive ability in a contemporary Swedish cohort. At birth, 50% of the infants born at 24 to <31 weeks of gestation were predicted to have low risk of severe ROP and could potentially be released from ROP screening examinations. All routinely screened treated infants, excluding those screened for clinical indications of severe illness, were correctly flagged as needing ROP screening.

**Key messages**

**What is already known on this topic**

- DIGIROP decision support tool was developed using easy-to-obtain variables to identify infants predicted to not require retinopathy of prematurity (ROP) treatment and therefore might be released from all or some ROP examinations.

**What this study adds**

- This study confirms prediction ability on a new Swedish cohort.
- Medically complex infants should be excluded from the evaluation by the DIGIROP decision support tool and screened routinely.

**How this study might affect research, practice or policy**

- This study is a prerequisite for applying the DIGIROP support tool in centres with neonatal and ophthalmological settings similar to those in Sweden.

## Introduction

Retinopathy of prematurity (ROP) is a neurovascular vision-threatening disease that varies in incidence and severity worldwide.^1^ Infants with extreme prematurity and other neonatal morbidities are at higher risk, as are infants born in countries with inadequate neonatal intensive care, including poor control of oxygen.^1–3^ To detect ROP needing treatment, prematurely born babies undergo ROP screening examinations regularly, scheduled according to their gestational age (GA) at birth, postnatal (PNA) and postmenstrual (PMA) age and birth weight (BW).^4^ Currently, in Sweden, based on a recently updated guideline, all infants born at GA <30 weeks are screened, as well as severely ill infants requiring screening due to their increased ROP risk because of medical conditions.^5^ During the 10-year period 2008–2017 in Sweden, ~46 000 examinations were performed among ~7200 infants; only 6.1% required ROP treatment.^5^ Hence, there is a potential for optimisation of the ROP screening procedures to identify the small number of infants at risk of developing severe stages of ROP, as well as to identify those with the lowest risk for whom unnecessary examinations might be avoided. Safe and well-validated prediction models might be used for this purpose. Rigorous external validations of such models, continuously performed on temporally different cohorts, and countries with varying levels of neonatal intensive care, are crucial.

Several prediction models for various stages of ROP have been published. Many models include postnatal weight gain since poor weight gain is a known risk factor for severe ROP. The first such published model is the Weight, Insulin-like growth factor-1, ROP (WINROP) algorithm.^6 7^ Others are Children’s Hospital of Philadelphia ROP, the Colorado ROP model, Omaha ROP and the Postnatal Growth and ROP study (G-ROP).^8-11^ Weight at specific postnatal days, however, is not always available to the screening ophthalmologists for all infants but necessary for the models noted above. Therefore, we sought to construct a ROP prediction tool using easily obtainable variables. It resulted in two published prediction models for infants born at GA 24–30 weeks: an early estimating risk model for ROP treatment (DIGIROP-Birth) using only birth characteristics, and a second consisting of a set of risk models (DIGIROP-Screen) additionally using the timing for the first ROP diagnosis in their algorithms throughout the screening.^8 9^ A clinical decision support tool was suggested with the primary aim to identify low-risk infants that might be released from ROP examinations either at birth or later during the screening. Internal and external validations were performed in the original publications on a temporally different Swedish cohort, a German cohort and two US cohorts with successful replication of predictive values.

The current study aimed to validate DIGIROP models together with the decision support tool on a contemporary Swedish cohort with the data collected during years 2018–2020.

## Materials and methods

The working process followed the Transparent Reporting of a multivariable prediction model for Individual Prognosis or Diagnosis statement (online supplemental eAppendix 1) and the Prediction model study Risk of Bias Assessment Tool (PROBAST) instrument (online supplemental eAppendix 2).^10–12^

10.1136/bjophthalmol-2021-320738.supp1 Supplementary data

10.1136/bjophthalmol-2021-320738.supp2 Supplementary data

### Study population

Infants born at 24 to <31 weeks of gestation, from 8 August 2018 to 31 December 2019, and during 2020 at GA 24 to <30 weeks (due to new national guidelines), with completed and validated ROP screening data were included in the Swedish Contemporary Validation Cohort 2018–2020. For years 2018–2019, the data originated from the Swedish National Registry for ROP (SWEDROP) (n=981). For year 2020, the data were retrieved only for the Västra Götaland and Skåne region that had validated data available until the end of 2020 (n=216). In total, 1082 (882 from the SWEDROP and 200 from the two Swedish regions) out of 1197 (90.4%) infants were eligible for this study, of whom 57 (5.3%) were treated for ROP. During the study period, there were 61 (5.1%) infants born <24 weeks of GA, of whom 24 (39.3%) were treated for ROP, and 54 (4.5%) born ≥31 weeks of GA (none was treated for ROP, and all had severe medical issues). Due to inclusion criteria of the DIGIROP models, these 115 infants were excluded from the study. There was no missing data for the model input variables.

The Swedish Development Cohort 2007–2017, used to compare infants’ characteristics to the current validation cohort, was described elsewhere.^9^

### Study procedures

The fetal ultrasound determined GA. The PNA, PMA and GA were defined according to the American Academy of Pediatrics policy.^13^ BW SD scores (BWSDS) were standardised for GA and sex according to the Swedish 1990–1999 reference based on 800 000 healthy singletons.^14^ Data on comorbidities and parenteral nutrition for infants of specific interest were retrieved from the medical records.

### Study outcome and predictors

The studied outcome was ROP treatment performed according to the Early Treatment for ROP criteria or based on the examining ophthalmologist’s judgement.^15^ The International Classification of ROP was used for the definition of ROP severity.^16^ Predictors used for DIGIROP-Birth risk estimations were: GA (weeks and days), BW re-calculated to BWSDS in the algorithm and sex. The contribution of BWSDS in the model accounts for the infants’ immaturity, since BWSDS is the standardised difference between an infant’s weight and the mean weight of infants with same sex and GA at birth. DIGIROP-Screen required DIGIROP-Birth risk estimates, the status of (yes/no) and the age at the first sign of ROP continuously over time as input variables. The infants were followed from birth until regression of eventual ROP, with or without treatment, or until fully vascularised retina in case of no ROP.

### Statistical analysis

Continuous variables were presented by mean and SD or median and IQR, and categorical variables by number and percentage. For testing between the validation and the development cohorts, following tests were used: Fisher’s exact test (dichotomous variables), Mantel-Haenszel χ^2^ trend test (ordered categorical variables) and Mann-Whitney U test (continuous variables). All tests were two-tailed, and p<0.05 was considered statistically significant.

There were no differences from the development data in setting, eligibility criteria, outcome and predictors in this validation study. The estimated risk predictions based on DIGIROP-Birth represent early risk for ROP treatment. DIGIROP-Screen risk estimates for ROP treatment were calculated at PNA 6, 7, 8, 9, 10, 11, 12, 13 and 14 weeks. The algorithms will be available at a free-of-charge online application.^17^ Data were analysed on the patient level considering the most severely affected eye. GA-specific cut-offs, constructed based on the model development cohort to achieve 100% sensitivity, were used for validation of the clinical decision support tool.^9^ The model’s generalisability/transportability on this Swedish contemporary validation cohort was evaluated by sensitivity, specificity, cumulative specificity (along the screening for DIGIROP-Screen), positive predictive value (PPV), negative predictive value (NPV), model accuracy and area under the receiver operating characteristic (ROC) curve (AUC) with 95% CI. Cumulative specificity is the proportion of infants suggested by the tool to not require screening (at the current time point or any time before) who did not actually require treatment. Details about sample size considerations, and calculations of DIGIROP-Birth and DIGIROP-Screen risk estimates are presented in the online supplemental eAppendix 3.

10.1136/bjophthalmol-2021-320738.supp3 Supplementary data

Analyses were performed using SAS software V.9.4 (SAS Institute, Cary, North Carolina, USA).

## Results

### Study population

Infants’ characteristics are presented in table 1. Among 1082 included infants, 484 (44.7%) were girls; mean GA was 28.2 (SD 1.9) weeks; 28.3 (SD 1.8) weeks for infants without ROP treatment and 25.3 (SD 1.1) weeks for infants with ROP treatment. Mean BW was 1117 (SD 340) g overall, standardised BW adjusted for GA and sex, BWSDS, −1.08 (SD 1.41) for infants with no treatment compared with −1.75 (SD 1.98) for those with ROP treatment. Any ROP was diagnosed in 338 (31.2%) infants, with approximately one-third each having maximum ROP stage 1, 2 and 3. Median PNA at first ROP diagnosis was 8.1 (IQR 6.9–9.7) weeks. Median PNA at first ROP treatment, among 57 (5.3%) infants that received treatment, was 12.6 (IQR 11.0–13.6) weeks. All infants with ROP treatment were born at ≤28 weeks of GA. Infants with no ROP treatment had a median of 4 (IQR 3–7, sum 5980) ROP screening examinations, and those requiring treatment had a median of 17 (IQR 14–22, sum 1012) examinations.

**Table 1:** Infants’ characteristics at birth, first sign of ROP, maximum stage and ROP treatment (Swedish Contemporary Validation Cohort 2018–2020)

Variable	Total (n=1082*)	No ROP treatment (n=1025)	ROP treatment (n=57)
Sex			
Boy	598 (55.3%)	570 (55.6%)	28 (49.1%)
Girl	484 (44.7%)	455 (44.4%)	29 (50.9%)
Gestational age at birth (weeks)	28.2 (1.9)	28.3 (1.8)	25.3 (1.1)
Gestational age (full weeks)			
24	71 (6.6%)	44 (4.3%)	27 (47.4%)
25	99 (9.1%)	83 (8.1%)	16 (28.1%)
26	131 (12.1%)	124 (12.1%)	7 (12.3%)
27	154 (14.2%)	148 (14.4%)	6 (10.5%)
28	165 (15.2%)	164 (16.0%)	1 (1.8%)
29	247 (22.8%)	247 (24.1%)	0 (0.0%)
30	215 (19.9%)	215 (21.0%)	0 (0.0%)
Birth weight (g)	1117 (340)	1142 (329)	667 (185)
Birth weight SDS	−1.11 (1.46)	−1.08 (1.41)	−1.75 (1.98)
Maximum ROP stage			
No ROP	744 (68.8%)	744 (72.6%)	0 (0.0%)
ROP stage 1	122 (11.3%)	122 (11.9%)	0 (0.0%)
ROP stage 2	107 (9.9%)	104 (10.1%)	3 (5.3%)
ROP stage 3	106 (9.8%)	55 (5.4%)	51 (89.5%)
ROP stage 4B	3 (0.3%)	0 (0.0%)	3 (5.3%)
Postnatal weeks to first ROP diagnosis	8.1 (6.9; 9.7) n=338	8.0 (6.6; 9.7) n=281	8.4 (7.6; 10.0) n=57
Postnatal weeks to first ROP treatment			12.6 (11.0; 13.6)
Number of ROP screening examinations	5 (3; 8)	4 (3; 7)	17 (14; 22)
DIGIROP-Birth risk estimate (probability)	0.006 (0.001; 0.045)	0.005 (0.001; 0.033)	0.239 (0.108; 0.321)

For categorical variables, n (%) is presented.

For continuous variables, mean (SD) or median (IQR) are presented.

*882 infants originate from the SWEDROP for years 2018–2019, and 200 infants only from the Västra Götaland and Skåne regions for year 2020.

ROP, retinopathy of prematurity; SDS, SD score; SWEDROP, Swedish National Registry for ROP.

Comparison of infants’ characteristics between the Swedish Contemporary Validation Cohort 2018–2020 and the Swedish Development Cohort 2007–2017 is provided in online supplemental table 1. Due to the screening guidelines’ changes from January 2020 (from previously <31 weeks to <30 weeks of GA), the infants had lower GA, lower weight at birth and higher DIGIROP-Birth risk estimates in the validation cohort than in the development cohort.

### Validation of DIGIROP-Birth and its decision support tool on a Swedish Contemporary Validation Cohort 2018–2020

**Figure 1:**
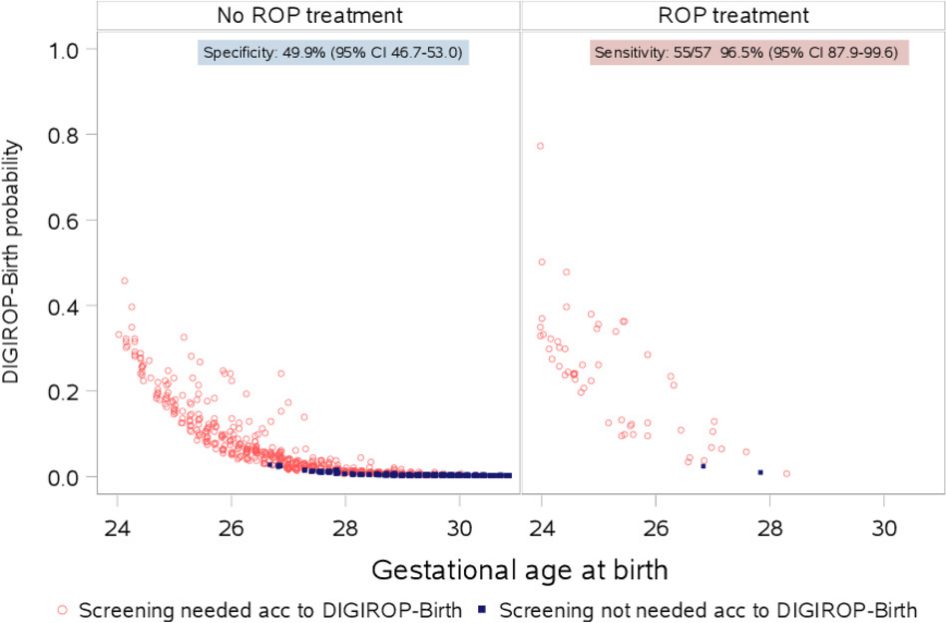
DIGIROP-Birth risk estimates and achieved sensitivity and specificity (Swedish Contemporary Validation Cohort 2018–2020). ROP, retinopathy of prematurity.

Median DIGIROP-Birth risk probabilities were 0.005 (IQR 0.001–0.033) for infants not requiring ROP treatment and 0.239 (IQR 0.108–0.321) for those with at least one ROP treatment session (table 1). A scatter plot presenting all DIGIROP-Birth probabilities is shown in figure 1.

The AUC was 0.93 (95% CI 0.90 to 0.95) (table 2). After applying GA-specific cut-offs, originally identified to achieve 100% sensitivity, the sensitivity in the current cohort was 96.5% (95% CI 87.9 to 99.6), specificity 49.9% (95% CI 46.7 to 53.0), PPV 9.7% (95% CI 7.4 to 12.4), NPV 99.6% (95% CI 98.6 to 100). Considering both true positives and true negatives, the calculated model accuracy was 52.3% (95% CI 49.3 to 55.3). In the subgroup of infants born at GA <30 weeks, the specificity was 36.8% (95% CI 33.5 to 40.2).

**Table 2:** Sensitivity, specificity, cumulative specificity, positive predictive value, negative predictive value, model accuracy and area under the receiver operating characteristic curve with 95% CI for DIGIROP-Birth and DIGIROP-Screen (Swedish Contemporary Validation Cohort 2018–2020)

	Sensitivity		Specificity	Cumulative specificity	Positive predictive value	Negative predictive value	Model accuracy	Area under the ROC curve
Model and time point	n/N*	% (95% CI)	% (95% CI)	% (95% CI)	% (95% CI)	% (95% CI)	% (95% CI)	AUC (95% CI)
**DIGIROP-Birth**	55/57†‡	96.5 (87.9 to 99.6)	49.9 (46.7 to 53.0)	49.9 (46.7 to 53.0)	9.7 (7.4 to 12.4)	99.6 (98.6 to 100.0)	52.3 (49.3 to 55.3)	0.93 (0.90 to 0.95)
**DIGIROP-Screen PNA6w**	55/57†‡	96.5 (87.9 to 99.6)	48.9 (45.8 to 52.0)	50.0 (46.8 to 53.1)	9.5 (7.2 to 12.2)	99.6 (98.6 to 100.0)	51.4 (48.4 to 54.4)	0.93 (0.90 to 0.95)
**DIGIROP-Screen PNA7w**	55/57†‡	96.5 (87.9 to 99.6)	48.1 (45.0 to 51.2)	50.9 (47.8 to 54.0)	9.4 (7.1 to 12.0)	99.6 (98.5 to 100.0)	50.6 (47.6 to 53.7)	0.93 (0.90 to 0.96)
**DIGIROP-Screen PNA8w**	54/56†‡	96.4 (87.7 to 99.6)	54.3 (51.2 to 57.4)	57.4 (54.3 to 60.4)	10.3 (7.9 to 13.3)	99.6 (98.7 to 100.0)	56.5 (53.5 to 59.5)	0.93 (0.90 to 0.96)
**DIGIROP-Screen PNA9w**	52/54†‡	96.3 (87.3 to 99.5)	62.8 (59.8 to 65.8)	66.0 (63.0 to 68.9)	12.0 (9.1 to 15.4)	99.7 (98.9 to 100.0)	64.5 (61.6 to 67.4)	0.94 (0.91 to 0.96)
**DIGIROP-Screen PNA10w**	51/52†	98.1 (89.7 to 100.0)	67.0 (64.1 to 69.9)	70.2 (67.3 to 73.0)	13.1 (9.9 to 16.9)	99.9 (99.2 to 100.0)	68.5 (65.7 to 71.3)	0.94 (0.91 to 0.96)
**DIGIROP-Screen PNA11w**	40/43‡§¶	93.0 (80.9 to 98.5)	70.0 (67.1 to 72.8)	72.8 (69.9 to 75.5)	11.5 (8.4 to 15.4)	99.6 (98.8 to 99.9)	71.0 (68.1 to 73.7)	0.94 (0.91 to 0.96)
**DIGIROP-Screen PNA12w**	29/31‡§	93.5 (78.6 to 99.2)	70.0 (67.1 to 72.8)	72.8 (69.9 to 75.5)	8.6 (5.9 to 12.2)	99.7 (99.0 to 100.0)	70.7 (67.9 to 73.5)	0.94 (0.91 to 0.96)
**DIGIROP-Screen PNA13w**	24/25‡	96.0 (79.6 to 99.9)	77.0 (74.3 to 79.5)	78.7 (76.1 to 81.2)	9.2 (6.0 to 13.4)	99.9 (99.3 to 100.0)	77.4 (74.8 to 79.9)	0.94 (0.92 to 0.96)
**DIGIROP-Screen PNA14w**	10/10	100.0 (69.2 to 100.0)	76.3 (73.6 to 78.9)	78.7 (76.1 to 81.2)	4.0 (1.9 to 7.1)	100.0 (99.5 to 100.0)	76.5 (73.8 to 79.1)	0.97 (0.95 to 0.99)

*Infants are followed up until their first ROP treatment, why the number of patients at risk among those requiring treatment in the future (N) decreases along the screening.

†Severe medical condition (intraventricular haemorrhage, hydrocephalus), ROP treatment stage 3, zone II–III, pre-plus disease.

‡Syndrome (Beckwith-Wiedemann), ROP treatment zone II–III, plus disease right eye, pre-plus disease left eye.

§Severe medical condition (necrotising enterocolitis, small for gestational age, bronchopulmonary dysplasia), incomplete examinations (hazy eyes), ROP treatment stage 3, zone II–III, pre-plus disease.

¶Severe medical condition (small for gestational age, iatrogen chylothorax), incomplete (hazy eyes) and missed examinations, ROP treatment stage 3 zone II, plus disease.

AUC, area under the curve; PNA, postnatal age; ROC, receiver operating characteristic; w, weeks.

All children with severe medical issues should be evaluated with screening. Two infants were inappropriately flagged as not needing ROP screening. One of them was diagnosed with Beckwith-Wiedemann syndrome, and was treated according to criteria. The other infant had intraventricular haemorrhage grade IV and hydrocephalus and was treated for ROP stage 3, zone II–III, pre-plus disease (ie, did not fulfil treatment criteria). The number of days with parenteral nutrition for those infants was 25 and 58 days, respectively.

**Figure 2:**
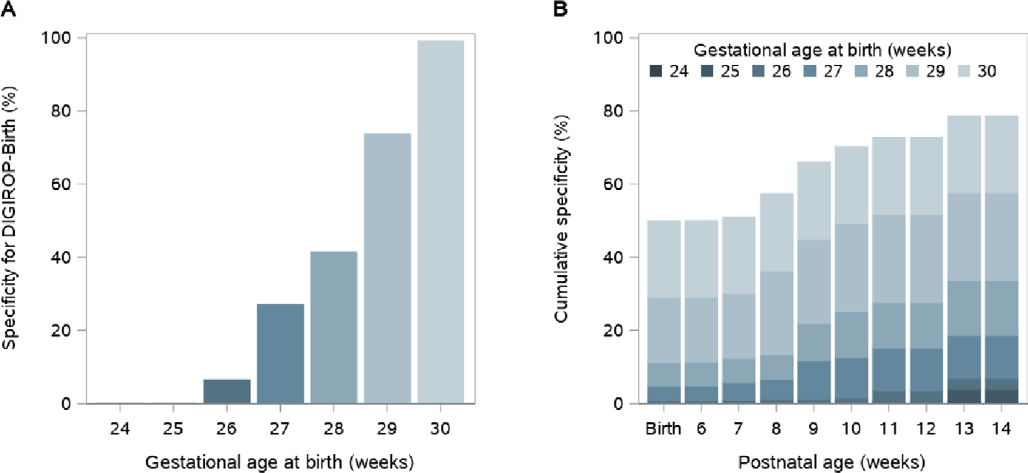
Specificity and cumulative specificity for DIGIROP decision support tool, (A) by gestational age at birth based on DIGIROP-Birth and (B) by postnatal age based on DIGIROP-Birth and DIGIROP-Screen (Swedish Contemporary Validation Cohort 2018–2020).

All infants born at GA 24 and 25 weeks were flagged for ROP screening using DIGIROP-Birth. It was predicted that some slightly higher GA infants could potentially be released from ROP screening: 8 (6.5%) infants with GA 26 weeks at birth, 40 (27.0%) with GA 27 weeks, 68 (41.5%) with GA 28 weeks, 182 (73.7%) with GA 29 weeks and 213 (99.1%) with GA 30 weeks, according to the model among infants with no ROP treatment (figure 2A). In total, 2018/5980 (33.7%) of all ROP screening examinations among non-treated infants could potentially have been avoided. Concerning only infants born at GA <30 weeks, that is, the present Swedish guidelines, applying DIGIROP-Birth would save 1313/5265 (24.9%) ROP examinations.

### Validation of DIGIROP-Screen and its decision support tool on a Swedish Contemporary Validation Cohort 2018–2020

Estimated individual probabilities for not treated and ROP-treated infants for PNA 6–14 weeks are presented in online supplemental figure 1A-I.

**Figure 3:**
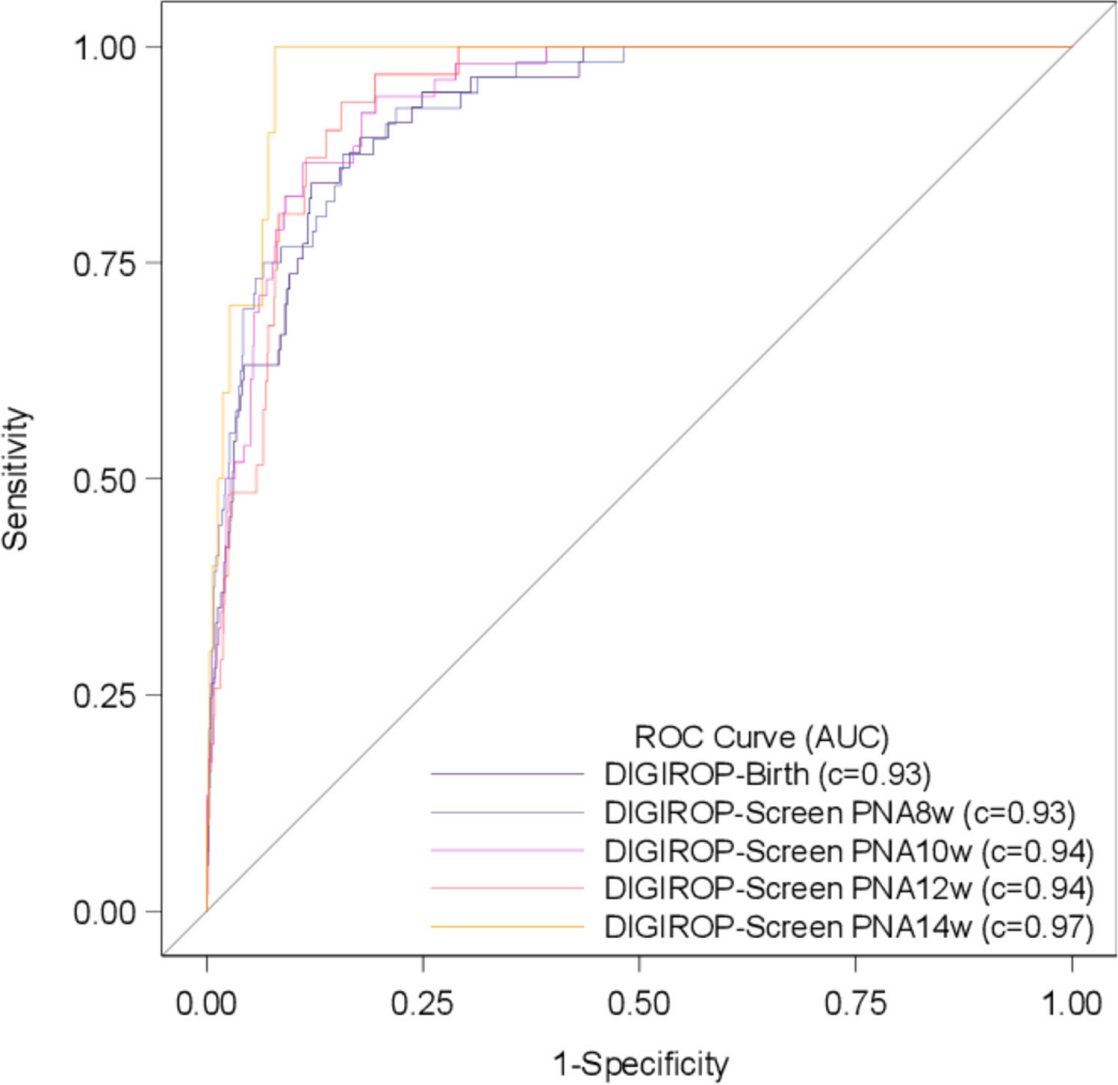
Receiver operating characteristic (ROC) curves for estimates obtained by DIGIROP-Birth and DIGIROP-Screen at PNAs 8, 10, 12 and 14 weeks (Swedish Contemporary Validation Cohort 2018–2020). AUC, area under the curve; PNA, postnatal age.

The AUCs ranged between 0.93 and 0.97 for different models of DIGIROP-Screen PNA 6–14 weeks (table 2). The sensitivity ranged between 93.0% and 100%. The cumulative specificity increased from 50.0% (95% CI 46.8 to 53.1) at PNA 6 weeks to 78.7% (95% CI 76.1 to 81.2) at PNA 14 weeks; among infants born at GA <30 weeks from 36.9% (95% CI 33.6 to 40.3) at PNA 6 weeks to 73.1% (95% CI 69.9 to 76.1). The model accuracy ranged from 50.6% to 77.4% during the screening (table 2). The cumulative specificity by GA for different PNAs is presented in figure 2B. Selected ROC curves are presented in figure 3.

Additionally, two infants were flagged as not needing ROP screening but were treated for ROP. One, with necrotising enterocolitis, small for GA, bronchopulmonary dysplasia and incomplete examinations due to hazy eyes was treated for ROP stage 3, zone II–III, pre-plus disease, and was missed by the tool at PNA 11 and 12 weeks. However, treatment was provided despite not fulfilled treatment criteria. The other infant, with severe medical conditions (small for GA, iatrogen chylothorax), had incomplete examinations due to hazy eyes, and missed examinations, was missed by the tool at PNA 11 weeks and was later treated for ROP after fulfilling treatment criteria. This infant would have been flagged needing screening if the baby was screened at PNA week 11 as was originally planned, and then was diagnosed with ROP. The number of days with parenteral nutrition for the two infants was 38 and 63 days, respectively.

## Discussion

In this validation study, performed on a contemporary Swedish cohort of 1082 infants born at 24–30 weeks of gestation, we found that DIGIROP-Birth, DIGIROP-Screen and their decision support tool had a similar model performance as that obtained on the Swedish Development Cohort.

The models had a high predictive ability with an AUC ranging between 0.93 and 0.97 depending on the time point evaluated. The specificity was 50% at birth and increased up to 79% cumulatively during the screening (37%–73% among infants born at GA <30 weeks), compared with 53%–81% obtained for the 6991 infants included in the development cohort, and 46%–75% in the external validation performed on a Swedish (n=314), German (n=322) and US (n=605) cohorts in the original publication.^9^ Specificity in this study stands for the percentage of low-risk infants never requiring ROP treatment correctly identified not needing ROP screening. Achieving specificity of 50% using only birth characteristics (GA, BW and sex) is noteworthy, considering that about 30% of all screened Swedish prematurely born infants develop any ROP.^5^ This means that according to the model, 50% of infants might be released from all ROP screening examinations, corresponding to one-third of all unnecessary examinations. The cumulative specificity increased to 79% during the screening, which means that 29% (79% cumulative specificity−50% specificity of DIGIROP-Birth) of by-the-tool-released infants required at least one ROP examination to get the probability for ROP treatment estimated and the tool applied. Based on an ongoing meta-analysis in our research group, mean costs per ROP screening examination in high-income economy countries were shown to range between US$106 and US$250, in total costing between US$213 908 and US$504 500 for the 2018 examinations saved.

The sensitivity ranged between 93% and 100%. Four of 57 infants were flagged as not needing ROP screening, two at birth and additionally two during the screening, although ROP treatment was given. However, only one of the four children was a true miss-flagged infant corresponding to 98% sensitivity for the model. All four infants had severe medical conditions, and none of them would have been released from the continuous follow-ups based on medical judgement. Given the data collected in this study, we recommend not to use DIGIROP decision support tool for infants diagnosed with severe congenital malformations/syndromes, hydrocephalus and for those that have performed intestinal surgery, for example, for necrotising enterocolitis. Additionally, the opthalmologist’s medical judgement should always take precedence over the DIGIROP decision support tool. Two of the four infants did not fulfil indication for treatment and hence were not true miss-flagged infants. However, we do not know if they would have developed treatment warranting ROP if not treated early. The two infants missed during the screening did not have regular or had incomplete screening examinations, potentially resulting in incorrect value for the timing of first sign of ROP. This highlights the importance of data validity awareness, regarding both lack of data and potentially incorrect data. Although not explicitly studied in the current work, poor interophthalmologist agreement regarding ROP classification has been previously reported.^18^ The increased use of image-based diagnostics will likely improve ROP data quality.^19^

Several prediction models have been published aiming to discriminate any ROP from no ROP, or type 1 ROP versus no type 1 ROP (ROP treatment vs no ROP treatment).^6 7 20–30^ The majority of those models require specifically timed longitudinal weights/weight gain that are not always available for all infants. Slidsborg *et al* published a Danish model for ROP treatment on 4182 infants including only GA and BW. They reported 100% sensitivity and the percentage of infants for whom number of examinations could be reduced or eliminated was 17%.^29^ Ying *et al* studied the predictive value of perinatal risk factors, Apgar score at 1 min, maternal race, birth location and intubation, on type 1 ROP (and any ROP) for 7483 included infants, concluding a minimal additional contribution besides GA and BW.^30^ This study did not present any cut-offs for decision support. To our knowledge, there are no other early estimating risk models for ROP treatment/type I ROP based solely on the birth characteristics other than the DIGIROP-Birth model. DIGIROP-Birth was conducted on ~7000 infants; in this validation study, the AUC was 0.93; based on its decision support tool, the sensitivity was 96% (98% considering infants with eligible data) and the specificity 50% for infants born at GA 24 to <31 weeks (37% for infants born at GA 24 to <30 weeks). Early risk estimation and identification of low-risk and high-risk infants might facilitate the planning of the neonatal healthcare resources and provide information about infants’ prognoses to their parents/guardians.

Until now, DIGIROP-Screen model, and the decision support tool have not been validated outside our research group. The risk of bias for the DIGIROP-Birth model according to the PROBAST instrument has been scrutinised by Zackula and Raghuveer.^31 32^ The model has been validated on a Portuguese cohort of 257 infants (23 treated) and on a Chinese cohort of 442 infants (93 treated).^33 34^ In the Chinese cohort, the AUC of 0.63 for DIGIROP-Birth was unacceptably low; a model with AUC of at least 0.70 or higher is considered to be an acceptable prediction model. The model performed well in infants with younger GA and with extremely low BW. Overall, the model performance improved if apnoea and intraventricular haemorrhage were taken into account. In both of these validation studies, the initially published cut-offs for the DIGIROP decision support tool were not implemented. Instead, cohort-specific cut-offs were investigated. In the Portuguese cohort, the AUC was 0.82 for DIGIROP-Birth risk estimates. Applying the published decision support tool cut-offs obtained on the Swedish Development Cohort, the sensitivity would be 78% and specificity 56%. However, updating the cut-offs to optimise sensitivity (100%) in this cohort a specificity of 40% could be obtained for all infants, and 24% for those born at GA <30 weeks. These figures might be compared with the ones obtained in a validation study of G-ROP and WINROP using the same cohort, predicting type I ROP for infants born at GA <30 weeks; sensitivity was 100% for both G-ROP and WINROP and specificity 9% and 7%, respectively.^35^

The strength of this study is the access to the Swedish Contemporary Validation Cohort consecutively followed between years 2018 and 2020. Continuous validation is a prerequisite for a prediction model to be useful in clinics. Access to quality registers in Sweden enables such work. Another strength is that GA, BW and sex were available for all infants, implying no selection bias in the studied cohort. The limitation is that data originate from a register and not from a controlled prospective study, being the reason for the unclear overall judgement for the risk of bias according to the PROBAST instrument. The overall judgement of applicability, risk of bias for participants and analysis were considered to be low. None of the items had evaluated high concerns. The access to the information about medical conditions for all infants would facilitate identifying subgroups for whom these models are more or less reliable. The limitations include that the tool at present is best applied to the Swedish population. Future work should include planning of a study covering implementation of the tool into the clinics, as well as to continue validating the tool in population data in Sweden and elsewhere. The models could be modified to include variable(s) measuring severe illness or a proxy. The parameter estimates for the models or the cut-offs for the tool may be modified for other populations after testing.

This validation study of DIGIROP-Birth, DIGIROP-Screen and their decision support tool on a contemporary Swedish cohort showed an overall model performance similar to that obtained in the original publication. According to the decision support tool, 50% of infants could be released from all ROP screening examinations. The sensitivity ranged between 93% and 100%. Further validations of the DIGIROP models are recommended, considering a potential update that could account for severe medical conditions to get even closer to the 100% sensitivity at all time points.

## Data Availability

Data may be obtained from a third party and are not publicly available.
