# Supplementary material for: Validation of DIGIROP models and decision support tool for prediction of treatment for retinopathy of prematurity on a contemporary Swedish cohort
Source: Br J Ophthalmol. 2022 Mar 11;107(8):1132–8. doi: 10.1136/bjophthalmol-2021-320738 (PMC10359565; doi:10.1136/bjophthalmol-2021-320738)
Supplement: Supplementary data [file bjophthalmol-2021-320738supp002.pdf]

PROBAST

(Prediction model study Risk Of Bias Assessment Tool)

Published in Annals of Internal Medicine (freely available):

1. PROBAST: A Tool to Assess the Risk of Bias and Applicability of Prediction Model Studies
2. PROBAST: A Tool to Assess Risk of Bias and Applicability of Prediction Model Studies: Explanation and Elaboration

What does PROBAST assess?

PROBAST assesses both the *risk of bias* and *concerns regarding applicability* of a study that evaluates (develops, validates or updates) a multivariable diagnostic or prognostic prediction model. It is designed to assess primary studies included in a systematic review.

Bias occurs if systematic flaws or limitations in the design, conduct or analysis of a primary study distort the results. For the purpose of prediction modelling studies, we have defined *risk of bias* to occur when shortcomings in the study design, conduct or analysis lead to systematically distorted estimates of a model's predictive performance or to an inadequate model to address the research question. Model predictive performance is typically evaluated using calibration, discrimination and sometimes classification measures, and these are likely inaccurately estimated in studies with high risk of bias. *Applicability* refers to the extent to which the prediction model from the primary study matches your systematic review question, for example in terms of the participants, predictors or outcome of interest.

A primary study may include the development and/or validation or update of more than one prediction model. A PROBAST assessment should be completed for each distinct model that is developed, validated or updated (extended) for making individualised predictions. Where a publication assesses multiple prediction models, only complete a PROBAST assessment for those models that meet the inclusion criteria for your systematic review. Please note that subsequent use of the term "model" includes derivatives of models, such as simplified risk scores, nomograms, or recalibrations of models.

PROBAST is not designed for all multivariable diagnostic or prognostic studies. For example, studies using multivariable models to identify predictors associated with an outcome but not attempting to develop a model for making individualised predictions are not covered by PROBAST.

PROBAST includes four steps.

Step	Task	When to complete
**1**	Specify your systematic review question(s)	Once per systematic review
**2**	Classify the type of prediction model evaluation	Once for each model of interest in each publication being assessed, for each relevant outcome
**3**	Assess risk of bias and applicability	Once for each development and validation of each distinct prediction model in a publication
**4**	Overall judgment	Once for each development and validation of each distinct prediction model in a publication

If this is your first time using PROBAST, we strongly recommend reading the detailed explanation and elaboration (E&E, see link above) paper and to check the examples on www.probast.org

Pivodic A, *et al. Br J Ophthalmol* 2023; 107:1132–1138. doi: 10.1136/bjophthalmol-2021-320738

Step 1: Specify your systematic review question

State your systematic review question to facilitate the assessment of the applicability of the evaluated models to your question. *The following table should be completed once per systematic review.*

Criteria	Specify your systematic review question
Intended use of model:	**Prediction of ROP treatment and identification of infants that might be released from the ROP screening examinations, either at birth or during the screening process.**
Participants *including selection criteria and setting:*	**All infants prematurely born at gestational age of 24+0 to 30+6 (weeks+days).**
Predictors *(used in prediction modelling), including types of predictors (e.g. history, clinical examination, biochemical markers, imaging tests), time of measurement, specific measurement issues (e.g., any requirements/ prohibitions for specialized equipment):*	**Gestational age at birth (weeks and days), sex, birth weight (calculated to birth weight SDS in the algorithm), and the timing of the first diagnosis of ROP.**
Outcome to be predicted:	**ROP treatment according to the Early Treatment for Retinopathy of Prematurity (ETROP) criteria.**

Step 2: Classify the type of prediction model evaluation

Use the following table to classify the evaluation as model development, model validation or model update, or combination. Different signalling questions apply for different types of prediction model evaluation. If the evaluation does not fit one of these classifications then PROBAST should not be used.

**Classify the evaluation based on its aim**			
**Type of prediction study**	**PROBAST boxes to complete**	**Tick as appropriate**	**Definition for type of prediction model study**
Development only	Development		Prediction model development without external validation. These studies may include internal validation methods, such as bootstrapping and cross-validation techniques.
Development and validation	Development and validation		Prediction model development combined with external validation in other participants in the same article.
Validation only	Validation	**X**	External validation of existing (previously developed) model in other participants.

This table should be completed once for each publication being assessed and for each relevant outcome in your review.

**Publication reference**	**Pivodic A, Hard AL, Lofqvist C, et al. Individual Risk Prediction for Sight-Threatening Retinopathy of Prematurity Using Birth Characteristics. JAMA Ophthalmol 2019:1-9. doi: 10.1001/jamaophthalmol.2019.4502 [published Online First: 2019/11/08]**

	**Pivodic A, Johansson H, Smith LEH, et al. Development and validation of a new clinical decision support tool to optimize screening for retinopathy of prematurity. Br J Ophthalmol 2021 doi: 10.1136/bjophthalmol-2020-318719 [published Online First: 2021/05/14]**
**Models of interest**	**DIGIROP-Birth and DIGIROP-Screen, with the decision support tool.**
**Outcome of interest**	**ROP treatment according to the Early Treatment for Retinopathy of Prematurity (ETROP) criteria.**

Step 3: Assess risk of bias and applicability

PROBAST is structured as four key domains. Each domain is judged for risk of bias (low, high or unclear) and includes signalling questions to help make judgements. Signalling questions are rated as yes (Y), probably yes (PY), probably no (PN), no (N) or no information (NI). All signalling questions are phrased so that "yes" indicates absence of bias. Any signalling question rated as "no" or "probably no" flags the potential for bias; you will need to use your judgement to determine whether the domain should be rated as "high", "low" or "unclear" risk of bias. The guidance document contains further instructions and examples on rating signalling questions and risk of bias for each domain.

The first three domains are also rated for concerns regarding applicability (low/ high/ unclear) to your review question defined above.

Complete all domains separately for each evaluation of a distinct model. Shaded boxes indicate where signalling questions do not apply and should not be answered.

**DOMAIN 1: Participants**			
**A. Risk of Bias**			
Describe the sources of data and criteria for participant selection: **Data originates from the Swedish National Registry for ROP (SWEDROP) years 2018-2019 and from two Swedish regions for year 2020. Infants born at gestational age 24 -<31 weeks are intended for inclusion.**			
		Dev	Val
1.1 Were appropriate data sources used, e.g. cohort, RCT or nested case-control study data?		**N/A**	**Y**
1.2 Were all inclusions and exclusions of participants appropriate?		**N/A**	**Y**
**Risk of bias introduced by selection of participants**	**RISK:** (low/high/unclear)	**N/A**	**low**
Rationale of bias rating: **Only infants born at gestational age 24-<31 weeks are included according to the selected population used for development of the models. Among those, there were no infants with missing data concerning the variables of interest in the current study.**			
**B. Applicability**			
Describe included participants, setting and dates: **All infants born at gestational age 24-<31 weeks that have finalized ROP screening examinations (and have reported validated data) at Swedish neonatology sites are included according to the selected population used for development of the models. None of the included infants in the current study were part of development and validation studies in the models' original publications. The current external validation aimed to validate the models on a Swedish contemporary cohort. This study includes infants born from August 8 2018 to December 31 2020.**			
**Concern that the included participants and setting do not match the review question**	**CONCERN:** (low/high/unclear)	**N/A**	**low**
Rationale of applicability rating: **The included participants and setting correspond exactly to the population used for the development of the models and for the aim of the current study.**			

DOMAIN 2: Predictors			
**A. Risk of Bias**			
List and describe predictors included in the final model, e.g. definition and timing of assessment:			
		Dev	Val
2.1 Were predictors defined and assessed in a similar way for all participants?		**N/A**	**unclear**
2.2 Were predictor assessments made without knowledge of outcome data?		**N/A**	**Y**
2.3 Are all predictors available at the time the model is intended to be used?		**N/A**	**Y**
**Risk of bias introduced by predictors or their assessment**	**RISK:** (low/high/unclear)	**N/A**	**unclear**
Rationale of bias rating: **The data are originating from the register for ROP data, and are continuously updated over time. The potential need for ROP treatment (the study outcome) is a variable filled out at the latest stage after the predictors. The predictors are few and evaluated in the same way for GA, sex and BW used in DIGIROP-Birth, but regarding the timing when ROP is diagnosed for the first time (used in DIGIROP-Screen) there are couple of uncertain points. We know from one of our previous studies that there are patients that cannot be examined as early as needed due to their retinas being too immature (7.5% of patients according to a study performed at one site). We also know from previous studies that some patients are too ill or missed to be invited to the site in time. These points affect the correct identification of the first time when ROP is diagnosed (that should come as near in time as possible when the ROP has firstly occurred) and might be the reason why a certain patient is miss-flagged by the model not needing ROP screening. However, the same was valid also for the development cohort as data originates from the same register where this detailed information about each visit is not available.**			
**B. Applicability**			
**Concern that the definition, assessment or timing of predictors in the model do not match the review question**	**CONCERN:** (low/high/unclear)	**N/A**	**low**
Rationale of applicability rating: **There is no such concern, since the definitions are exactly the same as those used in the development phase.**			

Pivodic A, *et al. Br J Ophthalmol* 2023; 107:1132–1138. doi: 10.1136/bjophthalmol-2021-320738

DOMAIN 3: Outcome

A. Risk of Bias

Describe the outcome, how it was defined and determined, and the time interval between predictor assessment and outcome determination:

The studied outcome was ROP treatment introduced according to the Early Treatment for ROP criteria or based on the examining ophthalmologist's judgement. Most predictors are the infants' characteristics at birth reported independently from the outcome. The predictor timing for the first sign of ROP is defined at ROP screening examinations but clearly occurs before the outcome.

		Dev	Val
3.1 Was the outcome determined appropriately?		**N/A**	**low**
3.2 Was a pre-specified or standard outcome definition used?		**N/A**	**low**
3.3 Were predictors excluded from the outcome definition?		**N/A**	**low**
3.4 Was the outcome defined and determined in a similar way for all participants?		**N/A**	**unclear**
3.5 Was the outcome determined without knowledge of predictor information?		**N/A**	**low**
3.6 Was the time interval between predictor assessment and outcome determination appropriate?		**N/A**	**low**
**Risk of bias introduced by the outcome or its determination**	**RISK:** (low/high/unclear)	**N/A**	**unclear**

Rationale of bias rating:

The uncertainty around the outcome is regarding the previously reported poor inter-ophthalmologist agreement regarding ROP classification, although not possible to be studied in our work. The increased use of image-based diagnostics will likely improve ROP data quality. Additionally, since ROP treatment in some rare cases is given based on the examining ophthalmologist's judgement, the definition is not completely clearly stated by the ETROP criteria.

B. Applicability

At what time point was the outcome determined:

The timing of the outcome is clearly defined during the ROP screening examinations after all predictors.

If a composite outcome was used, describe the relative frequency/distribution of each contributing outcome:

		Dev	Val
**Concern that the outcome, its definition, timing or determination do not match the review question**	**CONCERN:** (low/high/unclear)	**N/A**	**low**

Rationale of applicability rating:

There is no concern that the outcome's definition, timing or determination does not match the review question.

Pivodic A, *et al. Br J Ophthalmol* 2023; 107:1132–1138. doi: 10.1136/bjophthalmol-2021-320738

DOMAIN 4: Analysis

A. Risk of Bias

Describe numbers of participants, number of candidate predictors, outcome events and events per candidate predictor:

Number of participants in this external validation study is 1082, of whom 57 (5.3%) experienced the study outcome, ROP treatment. Number of candidate predictors is not applicable for the validation study, since the model is already developed and tested in this work.

Describe how the model was developed (for example in regards to modelling technique (e.g. survival or logistic modelling), predictor selection, and risk group definition):

Not applicable for this validation study.

Describe whether and how the model was validated, either internally (e.g. bootstrapping, cross validation, random split sample) or externally (e.g. temporal validation, geographical validation, different setting, different type of participants):

Not applicable for this validation study. The current work is an external validation of the existing model on a contemporary Swedish cohort.

Describe the performance measures of the model, e.g. (re)calibration, discrimination, (re)classification, net benefit, and whether they were adjusted for optimism:

Please see presented Results section.

Describe any participants who were excluded from the analysis:

No infants were excluded from the analyses due to missing or incomplete data. However, the inclusion criteria is infants born 24-<31 weeks of gestation.

Describe missing data on predictors and outcomes as well as methods used for missing data:

There were no missing data in this cohort needed for model input.

		Dev	Val
4.1 Were there a reasonable number of participants with the outcome?		Dev	Val
4.2 Were continuous and categorical predictors handled appropriately?		**N/A**	**low**
4.3 Were all enrolled participants included in the analysis?		**N/A**	**low**
4.4 Were participants with missing data handled appropriately?		**N/A**	**low**
4.5 Was selection of predictors based on univariable analysis avoided?		**N/A**	**N/A**
4.6 Were complexities in the data (e.g. censoring, competing risks, sampling of controls) accounted for appropriately?		**N/A**	**low**
4.7 Were relevant model performance measures evaluated appropriately?		**N/A**	**low**
4.8 Were model overfitting and optimism in model performance accounted for?		**N/A**	**N/A**
4.9 Do predictors and their assigned weights in the final model correspond to the results from multivariable analysis?		**N/A**	**N/A**
**Risk of bias introduced by the analysis**	**RISK:** (low/high/unclear)	**N/A**	**low**

Rationale of bias rating:

All questions above were carefully evaluated. The potential issue could have been the low number of events. However, taking into account that the studied population is rare, the studied outcome among the studied population is also rare, and the importance of the rapid validation of the model on an external contemporary cohort, the number of 57 events was considered satisfactory. However, we are aware that a number of about 100 events at least is recommended by the guidelines.

PROBAST – Version of 15/05/2019
For more information, please see www.probast.org

Step 4: Overall assessment

Use the following tables to reach overall judgements about risk of bias and concerns regarding applicability of the prediction model evaluation (development and/or validation) across all assessed domains.
Complete for each evaluation of a distinct model.

**Reaching an overall judgement about risk of bias of the prediction model evaluation**	
**Low risk of bias**	If all domains were rated low risk of bias.<br>If a prediction model was developed without any external validation, and it was rates as low risk of bias for all domains, consider downgrading to **high risk of bias**. Such a model can only be considered as low risk of bias, if the development was based on a very large data set and included some form of internal validation.
**High risk of bias**	If at least one domain is judged to be at **high risk of bias**.
**Unclear risk of bias**	If an unclear risk of bias was noted in at least one domain and it was low risk for all other domains.

**Reaching an overall judgement about applicability of the prediction model evaluation**	
**Low concerns regarding applicability**	If low concerns regarding applicability for all domains, the prediction model evaluation is judged to have **low concerns regarding applicability.**
**High concerns regarding applicability**	If high concerns regarding applicability for at least one domain, the prediction model evaluation is judged to have **high concerns regarding applicability.**
**Unclear concerns regarding applicability**	If unclear concerns (but no "high concern") regarding applicability for at least one domain, the prediction model evaluation is judged to have **unclear concerns regarding applicability** overall.

**Overall judgement about risk of bias and applicability of the prediction model evaluation**		
**Overall judgement of risk of bias**	**RISK:** (low/high/unclear)	unclear
Summary of sources of potential bias: **The judgement of *unclear* potential bias is set due to the fact that data originates from a register and not from a controlled prospective study. Additionally, given the fact about known poor inter-ophthalmologist agreement regarding diagnosing and classification of ROP, we cannot be assured that the timing for the first ROP diagnosis and indication for ROP treatment have been evaluated in the same way for all individuals.**		
**Overall judgement of applicability**	**RISK:** (low/high/unclear)	low
Summary of applicability concerns: **There is no concern about the population, predictors, outcome, definitions and analysis for the studied models. They correspond to their respective ones in the model development cohort.**		

Pivodic A, *et al. Br J Ophthalmol* 2023; 107:1132–1138. doi: 10.1136/bjophthalmol-2021-320738
