# Supplementary material for: Validation of DIGIROP models and decision support tool for prediction of treatment for retinopathy of prematurity on a contemporary Swedish cohort
Source: Br J Ophthalmol. 2022 Mar 11;107(8):1132–8. doi: 10.1136/bjophthalmol-2021-320738 (PMC10359565; doi:10.1136/bjophthalmol-2021-320738)
Supplement: Supplementary data [file bjophthalmol-2021-320738supp003.pdf]

Supplementary eAppendix 3, eFigures and eTables to:

Validation of DIGIROP models and decision support tool for prediction of treatment for retinopathy of prematurity on a contemporary Swedish cohort

Aldina Pivodic, Lois E.H. Smith Anna-Lena Hård, Chatarina Löfqvist, Ana C. Almeida, Abbas Al-Hawasi, Eva Larsson, Pia Lundgren, Birgitta Sunnqvist, Kristina Tornqvist, Agneta Wallin, Gerd Holmström, Lotta Gränse

Contents

eAppendix 3. Details to Statistical Analysis 3

eFigure 1. DIGIROP-Screen Risk Estimates and Achieved Sensitivity and Specificity for Models at A) Postnatal Age of 6 Weeks, B) Postnatal Age of 7 Weeks, C) Postnatal Age of 8 Weeks, D) Postnatal Age of 9 Weeks, E) Postnatal Age of 10 Weeks, F) Postnatal Age of 11 Weeks, G) Postnatal Age of 12 Weeks, H) Postnatal Age of 13 Weeks, I) Postnatal Age of 14 Weeks (Swedish Contemporary Validation Cohort 2018-2020). 4

eTable 1. Infants' Characteristics (Swedish Contemporary Validation Cohort 2018-2020 versus Swedish Development Cohort 2007-2017). 9

eAppendix 3. Details to statistical analysis

A priori sample size calculation was not performed. We wanted to rapidly carry out an external validation of the published models on a sufficiently large Swedish cohort before implementation into the clinics. The effective sample size is defined by the number of studied events. Having 57 ROP treatments would result in the lower 95% confidence limit being 94% for a 100% sensitivity, which was considered sufficient for this rare population and outcome.

The DIGIROP-Birth prediction model for ROP treatment was developed using Poisson regression for time-varying data (*Holford 1976, 1980*). This method enables access to the continuous hazard function, *h(t)*, that describes the momentary/instantaneous rate for the outcome ROP treatment, i.e. the conditional probability that the studied event will occur in a very small interval given that it has not occurred before divided by the width of the interval. The hazard function is calculated as $h(t) = e^{\beta 0 + \beta 1X1 + \beta 2X2 + \cdots + \beta kXk}$ where *β0* to *βk* are the models estimated parameters and *X1* to *Xk* the infant specific values for the variables included in the model (*Pivodic et al. JAMA Ophthalmol. 2020*). From the hazard function the survival function $S(t) = e^{-\int_0^t h(u)\,du}$, and its complement, the cumulative risk function F(t)=1-S(t), were estimated.

The DIGIROP-Screen prediction models at PNA 6-14 weeks for ROP treatment were developed using logistic regression. The predicted probabilities were calculated as: $1/(1+e^{-LC})$, where LC is the linear predictor for a vector X of explanatory variables, i.e. *LC = β0 + β1X1 + β2X2 +... + βkXk*; where *β0* to *βk* are the models estimated parameters and *X1* to *Xk* the infant specific values for the variables included in the model (*Pivodic et al. Br J Ophthalmol. 2021).*

eFigure 1. DIGIROP-Screen risk estimates and achieved sensitivity and specificity for models at A) postnatal age of 6 weeks, B) postnatal age of 7 weeks, C) postnatal age of 8 weeks, D) postnatal age of 9 weeks, E) postnatal age of 10 weeks, F) postnatal age of 11 weeks, G) postnatal age of 12 weeks, H) postnatal age of 13 weeks, I) postnatal age of 14 weeks (Swedish contemporary validation cohort 2018-2020).

A)

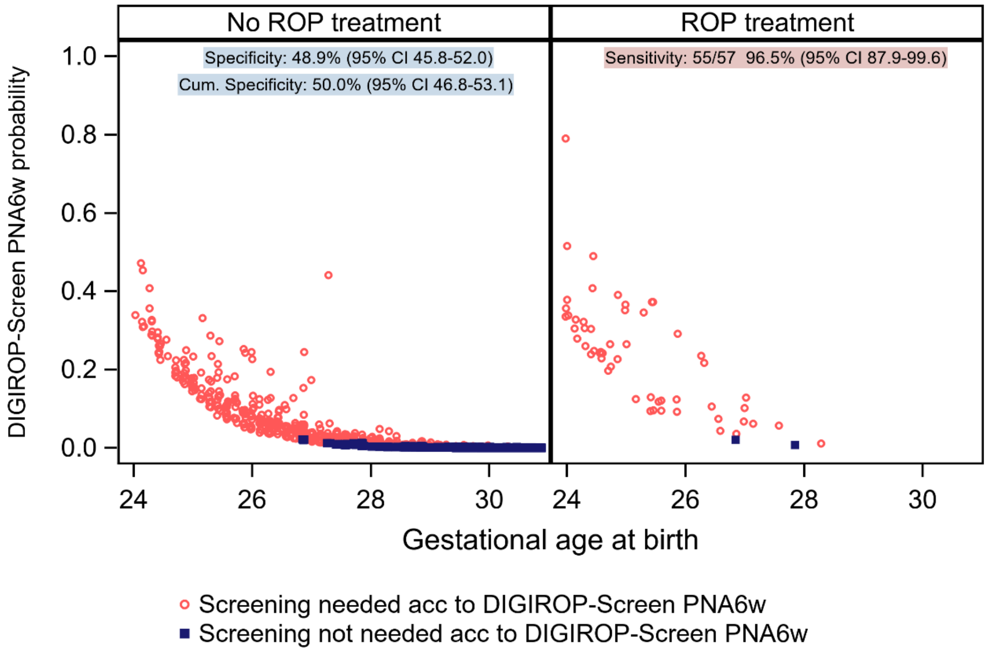

B)

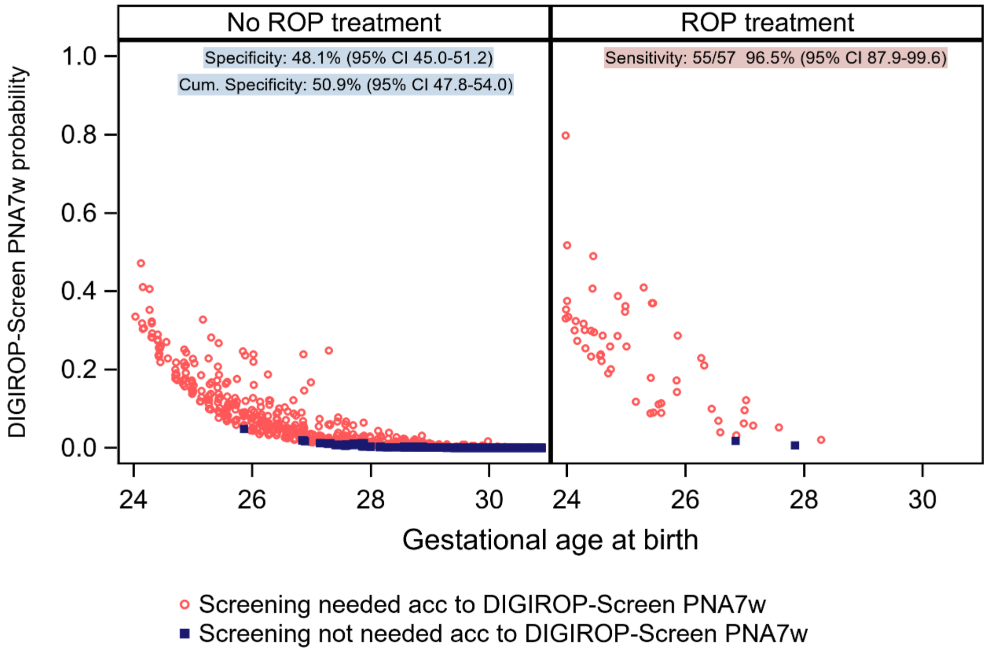

C)

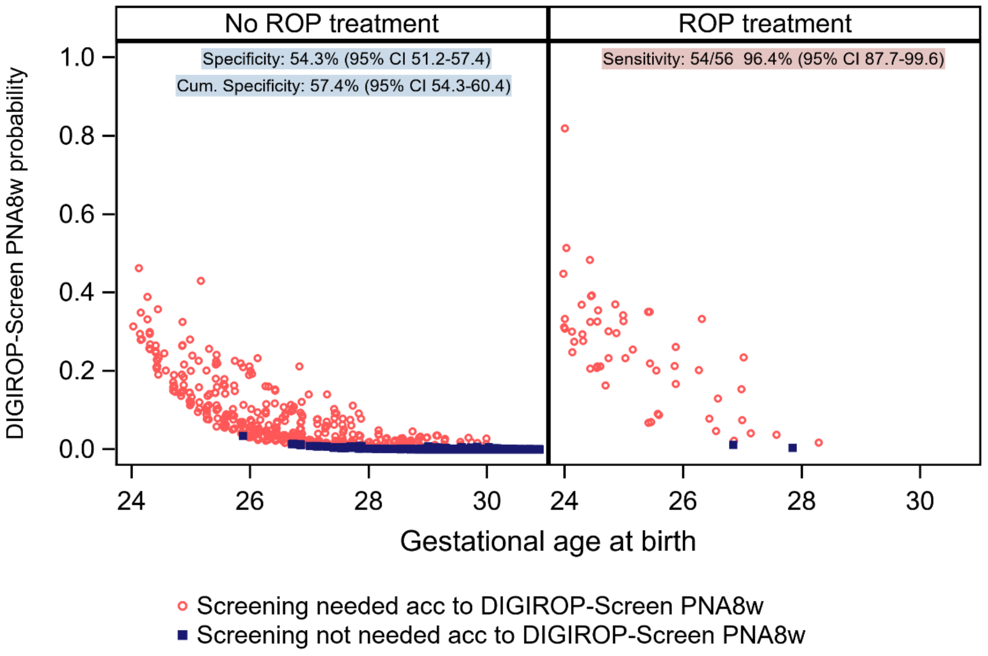
No ROP treatment
ROP treatment
Specificity: 54.3% (95% CI 51.2-57.4)
Cum. Specificity: 57.4% (95% CI 54.3-60.4)
Sensitivity: 54/56 96.4% (95% CI 87.7-99.6)
DIGIROP-Screen PNA8w probability
1.0
0.8
0.6
0.4
0.2
0.0
24
26
28
30
24
26
28
30
Gestational age at birth
Screening needed acc to DIGIROP-Screen PNA8w
Screening not needed acc to DIGIROP-Screen PNA8w

D)

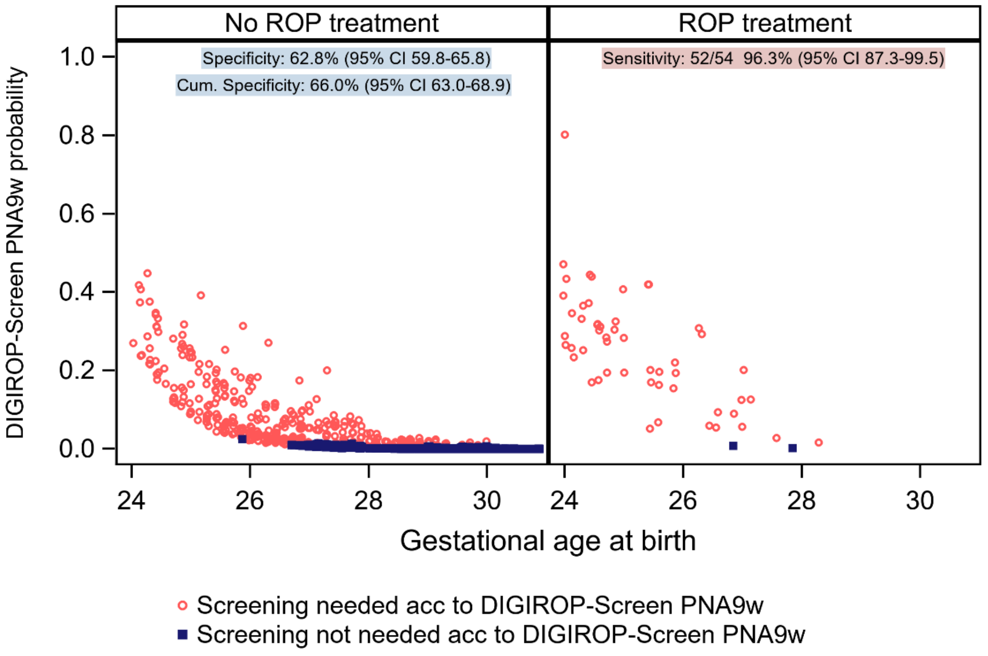
No ROP treatment
ROP treatment
Specificity: 62.8% (95% CI 59.8-65.8)
Cum. Specificity: 66.0% (95% CI 63.0-68.9)
Sensitivity: 52/54 96.3% (95% CI 87.3-99.5)
DIGIROP-Screen PNA9w probability
1.0
0.8
0.6
0.4
0.2
0.0
24
26
28
30
24
26
28
30
Gestational age at birth
Screening needed acc to DIGIROP-Screen PNA9w
Screening not needed acc to DIGIROP-Screen PNA9w

E)

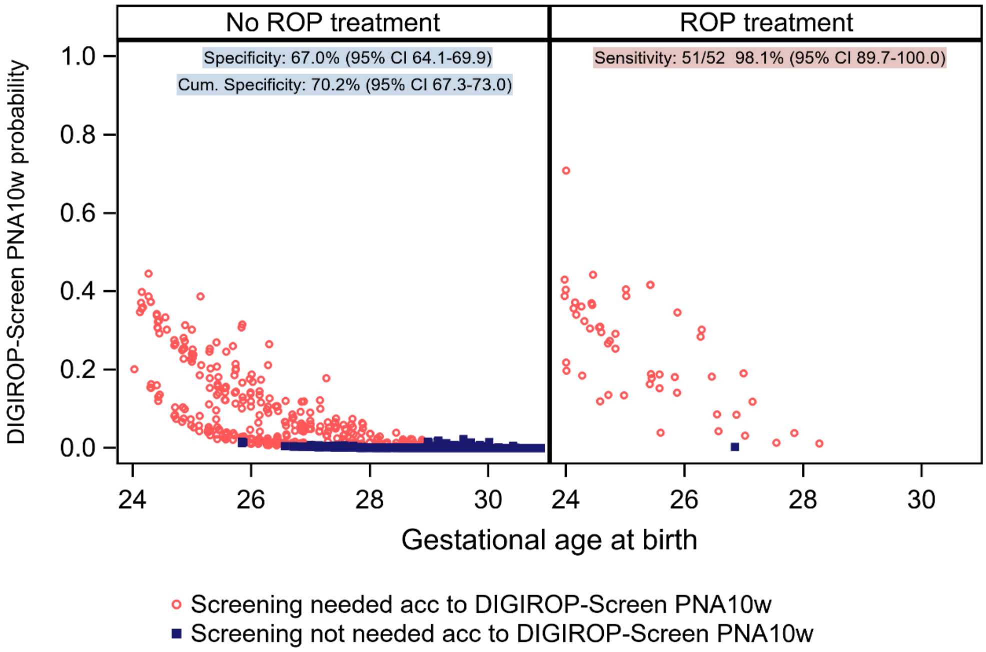
No ROP treatment
ROP treatment
Specificity: 67.0% (95% CI 64.1-69.9)
Cum. Specificity: 70.2% (95% CI 67.3-73.0)
Sensitivity: 51/52 98.1% (95% CI 89.7-100.0)
DIGIROP-Screen PNA10w probability
1.0
0.8
0.6
0.4
0.2
0.0
24
26
28
30
Gestational age at birth
Screening needed acc to DIGIROP-Screen PNA10w
Screening not needed acc to DIGIROP-Screen PNA10w

F)

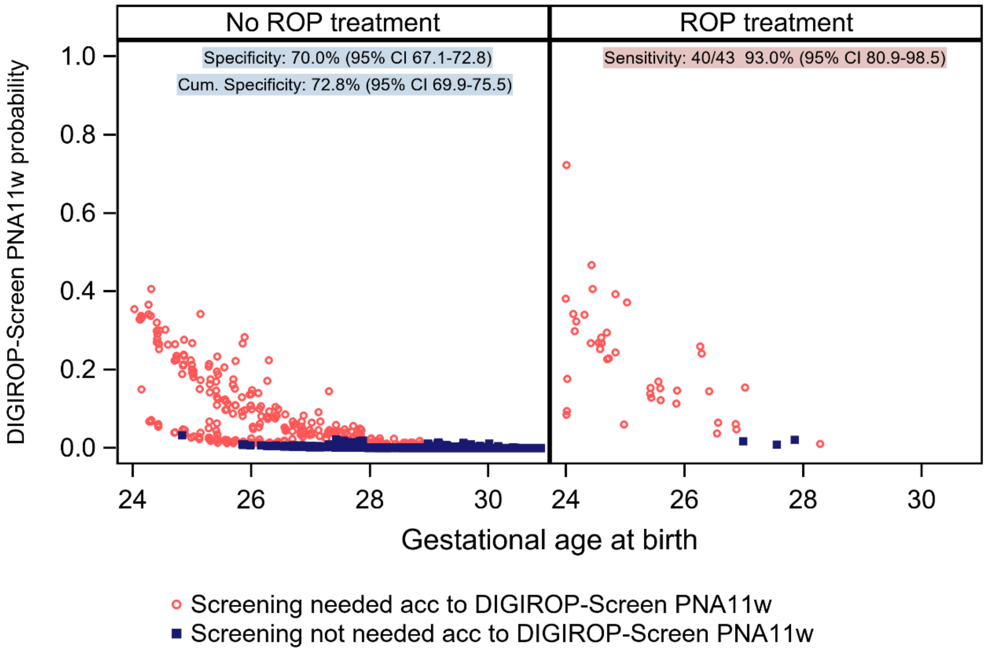
No ROP treatment
ROP treatment
Specificity: 70.0% (95% CI 67.1-72.8)
Cum. Specificity: 72.8% (95% CI 69.9-75.5)
Sensitivity: 40/43 93.0% (95% CI 80.9-98.5)
DIGIROP-Screen PNA11w probability
1.0
0.8
0.6
0.4
0.2
0.0
24
26
28
30
Gestational age at birth
Screening needed acc to DIGIROP-Screen PNA11w
Screening not needed acc to DIGIROP-Screen PNA11w

G)

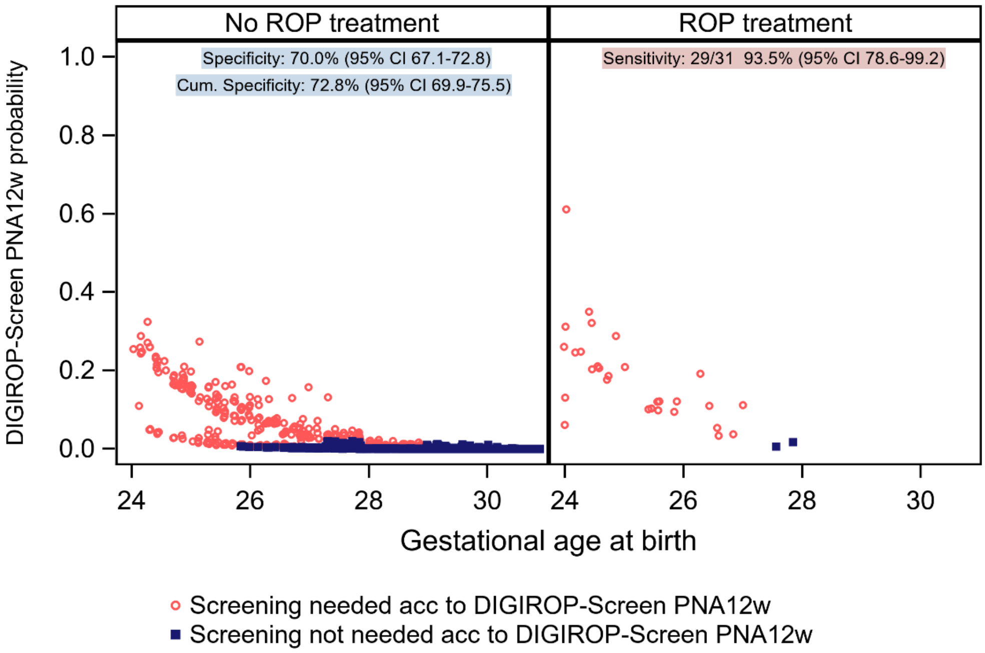
No ROP treatment
ROP treatment
Specificity: 70.0% (95% CI 67.1-72.8)
Cum. Specificity: 72.8% (95% CI 69.9-75.5)
Sensitivity: 29/31 93.5% (95% CI 78.6-99.2)
DIGIROP-Screen PNA12w probability
1.0
0.8
0.6
0.4
0.2
0.0
24
26
28
30
24
26
28
30
Gestational age at birth
Screening needed acc to DIGIROP-Screen PNA12w
Screening not needed acc to DIGIROP-Screen PNA12w

H)

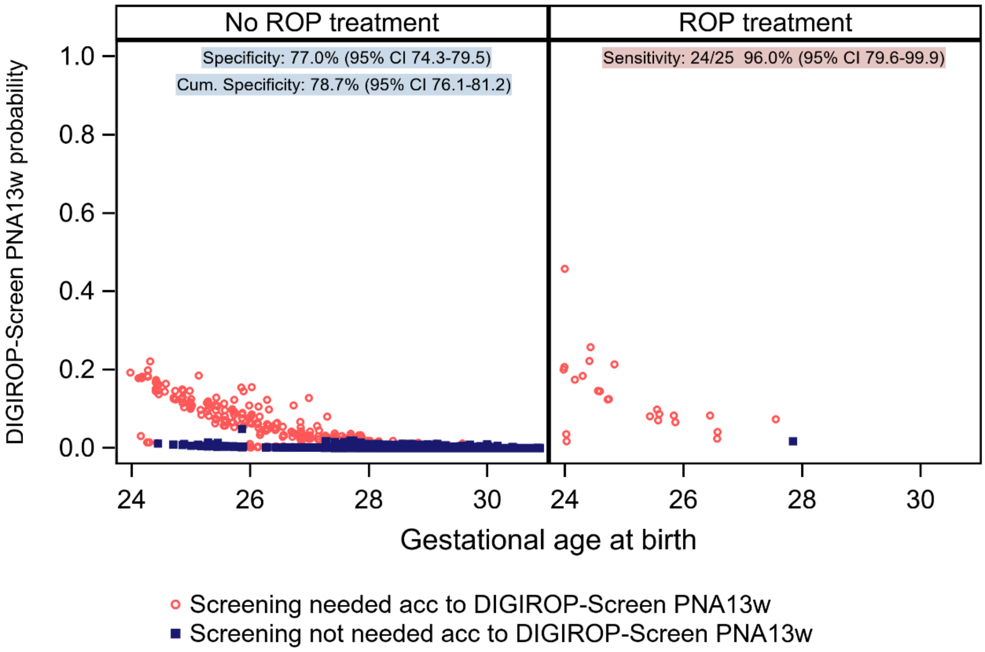
No ROP treatment
ROP treatment
Specificity: 77.0% (95% CI 74.3-79.5)
Cum. Specificity: 78.7% (95% CI 76.1-81.2)
Sensitivity: 24/25 96.0% (95% CI 79.6-99.9)
DIGIROP-Screen PNA13w probability
1.0
0.8
0.6
0.4
0.2
0.0
24
26
28
30
24
26
28
30
Gestational age at birth
Screening needed acc to DIGIROP-Screen PNA13w
Screening not needed acc to DIGIROP-Screen PNA13w

I)

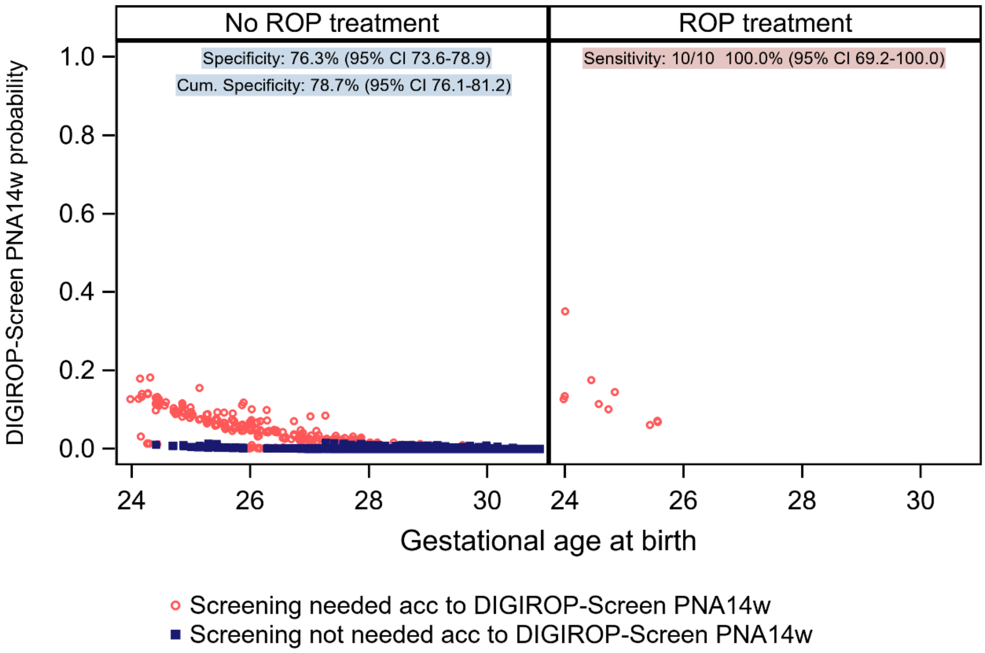
No ROP treatment
ROP treatment
Specificity: 76.3% (95% CI 73.6-78.9)
Cum. Specificity: 78.7% (95% CI 76.1-81.2)
Sensitivity: 10/10 100.0% (95% CI 69.2-100.0)
DIGIROP-Screen PNA14w probability
1.0
0.8
0.6
0.4
0.2
0.0
24
26
28
30
24
26
28
30
Gestational age at birth
Screening needed acc to DIGIROP-Screen PNA14w
Screening not needed acc to DIGIROP-Screen PNA14w

eTable 1. Infants' characteristics (Swedish contemporary validation cohort 2018-2020 versus Swedish development cohort 2007-2017).

Variable	Swedish Contemporary Validation Cohort 2018-2020 (n=1082)	Swedish Development Cohort 2007-2017 (n=6991)	p-value
**Sex**			
**Boy**	598 (55.3%)	3833 (54.8%)	
**Girl**	484 (44.7%)	3158 (45.2%)	0.81
**Gestational age at birth (weeks)**	28.2 (1.9)	28.3 (1.9)	0.024
**Gestational age at birth (full weeks)**			
**24**	71 (6.6%)	427 (6.1%)	
**25**	99 (9.1%)	597 (8.5%)	
**26**	131 (12.1%)	781 (11.2%)	
**27**	154 (14.2%)	914 (13.1%)	
**28**	165 (15.2%)	1141 (16.3%)	
**29**	247 (22.8%)	1419 (20.3%)	
**30**	215 (19.9%)	1712 (24.5%)	0.028
**Birth weight (g)**	1117 (340)	1146 (339)	0.012
**Birth weight SDS**	-1.11 (1.46)	-1.03 (1.37)	0.19
**Postnatal weeks to first ROP diagnosis**	8.1 (6.9; 9.7) n=338	8.1 (6.9; 9.7) n=2026	0.90
**ROP Treatment**	57 (5.3%)	287 (4.1%)	0.099
**Postnatal weeks to first ROP Treatment**	12.6 (11.0; 13.6) n=57	12.4 (10.9; 14.6) n=287	0.65
**DIGIROP-Birth risk estimate**	0.006 (0.001; 0.045)	0.005 (0.001; 0.04)	0.015

ROP = retinopathy of prematurity; SDS = standard deviation score; SD = standard deviation; IQR = interquartile range

For categorical variables n (%) is presented.
For continuous variables Mean (SD) or Median (IQR) are presented.
